# The Human Respiratory Virome in Health and Disease: Interactions, Dysbiosis, and Methodological Challenges

**DOI:** 10.1002/ggn2.202500022

**Published:** 2025-12-02

**Authors:** Xiaoxuan Yao, Xiaohui Zou, Bin Cao

**Affiliations:** ^1^ China‐Japan Friendship Hospital Chinese Academy of Medical Sciences & Peking Union Medical College Beijing China; ^2^ National Center for Respiratory Medicine State Key Laboratory of Respiratory Health and Multimorbidity National Clinical Research Center for Respiratory Diseases; Institute of Respiratory Medicine, Chinese Academy of Medical Sciences Department of Pulmonary and Critical Care Medicine, Center of Respiratory Medicine China‐Japan Friendship Hospital Beijing China; ^3^ New Cornerstone Science Laboratory, Department of Pulmonary and Critical Care Medicine, Center of Respiratory Medicine China‐Japan Friendship Hospital Beijing China

**Keywords:** virome, lung, respiratory disease

## Abstract

The human respiratory virome is an underexplored component of the microbiome that includes diverse DNA and RNA viruses such as eukaryotic viruses, bacteriophages, and archaeal viruses. Recent advances in metagenomics have revealed the complexity and dynamic nature of the human respiratory virome, which interacts closely with the host and the bacterial microbiome to influence respiratory health and disease. In healthy individuals, the virome is characterized by low biomass and high temporal variability, with *Anelloviruses* predominant in the upper airways, whereas *Streptococcus* phages and herpesviruses are most commonly detected in the lower airways. Common respiratory viruses, such as *respiratory syncytial virus*, *human rhinovirus*, and *influenza A virus*, can persist after acute infection and modulate host immunity. The respiratory virome also plays a significant role in chronic respiratory diseases. Despite its importance, research on the respiratory virome is hampered by technical challenges, including low viral abundance and limited reference databases. This review summarizes current understanding of the composition and determinants of the respiratory virome in healthy individuals, describes its interactions with the host and respiratory microbiota, including the potential modulatory roles of bacteriophages, outlines virome alterations in respiratory diseases, examines methodological challenges, and highlights potential clinical applications and future research directions.

## Graphical abstract

This article examines how the respiratory virome contributes to health and disease, highlighting its interactions with the host and bacteriome. Key disease associations and present knowledge gaps are outlined. The review discusses challenges in sample collection and preprocessing, and presents future research directions for understanding virome dynamics and improving respiratory disease management.

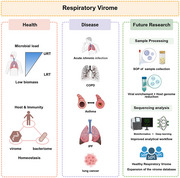

## 1 Introduction

The virome encompasses all viruses present within a given environment. In humans, the virome consists of eukaryotic viruses (infecting eukaryotic cells), bacteriophages (targeting human‐associated bacteria), and archaeal viruses (infecting archaea) [1, 2]. Its composition varies across anatomical sites due to distinct local microenvironments. Unlike bacteria and fungi, viruses do not possess universally conserved genomic regions such as the bacterial 16S or fungal 18S genes, which has impeded advances in virome research [3]. Nevertheless, recent technological developments—particularly next‐generation sequencing (NGS)—have enabled comprehensive characterization of the human virome [4]. The abundance and diversity of viral particles in the human body differ significantly depending on anatomical location. The gut, in particular, harbors the highest concentration and diversity of viruses, with approximately 10^9^ viral particles per gram of intestinal content [5]. The gut is a nutrient‐rich environment, characterized by a high density of epithelial cells and prokaryotic organisms, that supports extensive viral replication and exhibits age‐dependent diversity patterns [6, 7]. In contrast, other organs, especially the respiratory system, contain significantly fewer prokaryotic organisms. As a result, the phylogenetic composition of the virome and its impact on host health in these regions remain insufficiently characterized.

Emerging research demonstrates that even in healthy individuals, the respiratory tract contains a highly diverse viral community, comprising numerous commensal viruses that may contribute to microenvironmental stability and immune regulation [3, 5]. In‐depth research on the respiratory virome advances our understanding of the mechanisms underlying acute respiratory infections and provides new perspectives on the therapeutic strategies for chronic respiratory diseases [8, 9, 10]. Despite these recent advances, significant challenges remain in respiratory virome research. Compared to the gut virome, investigation on the respiratory virome remains in its infancy on a global scale, hindered by the difficulties associated with sample acquisition and technical constraints [11]. Current studies have predominantly focused on a narrow range of eukaryotic viruses known to cause respiratory illness, leaving the broader diversity of respiratory viruses—particularly in healthy individuals—largely unexplored [10, 11]. Additionally, given the low biomass characteristic of respiratory samples, the sensitive and accurate detection of low‐abundance viruses continues to present significant technical hurdles. From a data analysis perspective, distinguishing genuine viral signals from background noise and extracting biologically meaningful insights from complex viral interaction networks are ongoing methodological challenges that require novel bioinformatics algorithms.

In this review, we summarize recent progress in our understanding of the human respiratory virome and its roles in respiratory health and disease, while also highlighting the principal challenges that must be addressed to advance research in this field.

## 2 The Respiratory Virome in Health

### 2.1 The Upper Respiratory Tract (URT) Virome in Health

The respiratory system comprises upper and lower airways, with the URT extending from the nasal cavity to the larynx. This anatomical region is characterized by distinct structural features and specialized epithelial cells, directly interfacing with the external environment and is largely colonized by bacteria. The varying physiological conditions within the respiratory tract—including oxygen/carbon dioxide tension, pH, humidity, and temperature—create unique selective pressures that influence microbial colonization patterns [12]. These conditions establish a distinctive bacterial density gradient: peaking in the oropharynx (10^6^ bacteria/ml), declining through nasal regions (10^3^ bacteria/swab) and lungs (10^2^ bacteria/ml bronchoalveolar Lavage Fluid/BALF), while the environmental air exhibits only minimal bacterial presence (10^−1^ bacteria/cm^3^) [12]. Although similar systematic density gradients have not been established for viruses in the URT, this bacterial pattern demonstrates how physiological conditions can create distinct microbial niches along the respiratory tract.

Polymerase chain reaction (PCR)‐based studies reveal extensive viral presence in the URT, with distinct age‐dependent patterns. In children, respiratory viruses, including human rhinovirus, human bocavirus, polyomaviruses, and coronavirus, were detected in 67% of nasopharyngeal samples from 433 healthy children aged 6–24 months [13]. The asymptomatic adults (≥18 years) had much lower viral prevalence in nasopharyngeal and oropharyngeal swabs, with only 0.8% positive for rhinovirus and coronavirus, 0.4% for *Human metapneumovirus* (HMPV), and 2.1% for any respiratory virus [14]. However, another study of ambulatory adults over 65 years showed higher viral prevalence: 5.2% for rhinovirus, 2.4% for *Influenza A virus* (IAV), 2.6% for *Enterovirus*, 0.6% for *Respiratory syncytial virus* (RSV), and 9.3% for any virus [15]. These findings suggest that the detection rate of respiratory viruses in the upper respiratory tract is inconsistent and demonstrates a U‐shaped pattern with age, being higher in young children, lower in adults, and increasing again in older adults.

Metagenomic studies of the URT virome, primarily focusing on pediatric populations, have revealed deeper insights into viral composition. Studies from China [16], Europe [17], and the United States [18, 19] have shown that in nasal or nasopharyngeal swabs from healthy children, human viral sequences account for only 0.1–2.9% of total reads, with anelloviruses dominating across geographical regions. Secondary to *Anelloviridae* family, various viruses, including human herpesviruses (HHVs) [16, 18, 19], papillomaviruses [18], picornaviruses [18], and common respiratory viruses [16] such as human adenovirus, human rhinovirus, and IAV, were detected.

While respiratory microbiome research [20, 21] has made significant strides, our understanding of the URT virome, particularly its temporal evolution, remains limited. A landmark Mexican study has provided crucial insights into early‐life oropharyngeal virome development through a longitudinal investigation of nine asymptomatic children, followed from two weeks post‐birth through their first year [22]. Using monthly oropharyngeal swabs and metagenomic analysis, the study revealed viral presence as early as the first half‐week of life. The research uncovered a complex viral ecosystem comprising 50 distinct viral families, with five families (*Herpesviridae*, *Picornaviridae*, *Reoviridae*, *Virgaviridae*, and *Totiviridae*) constituting 95% of all viral sequence reads. At the species level, 50 different viruses were identified, including 15 human‐infecting and 27 plant‐infecting species. Among human‐infected viruses, *Cytomegalovirus* (CMV) showed universal presence with intriguing temporal patterns, while *Rotavirus A*, *Rhinovirus A*, and *Papillomavirus* were also frequently detected. The study observed a trend toward increasing human virus abundance across trimesters, though not reaching statistical significance. Neither Chao richness (a within‐sample/alpha richness estimator sensitive to rare taxa) nor Shannon diversity (a within‐sample/alpha diversity metric combining richness and evenness) showed significant changes across trimesters, suggesting complex rather than uniform URT virome development.

### 2.2 The Lower Respiratory Tract (LRT) Virome in Health

Traditional views held that healthy lungs should be sterile, with any microbial presence indicating a potential pathological state. However, modern molecular biology techniques have revealed that the healthy lung contains sparse but definite microbial communities. More importantly, these microbial communities exhibit characteristics of dynamic equilibrium—a state of constant flux where introduction and elimination of microbes occur simultaneously, maintaining overall stability despite ongoing changes— rather than static existence [12, 21]. The dynamic nature of the LRT microbiome stems from the continuous balance between microbial immigration and clearance. Two prominent models—adapted island model [23] and the neutral model [24] — have been constructed to describe the microbiome's biogeography in healthy lower airways. Specifically, the adapted island model suggests the lower airway microbiome is shaped by a balance between microbial migration from the URT (e.g., micro‐aspiration, mucosal dispersion) and clearance processes (e.g., coughing, ciliary movement), resulting in decreasing microbial density down the respiratory tract [23]. Similarly, the neutral model attributes lung microbiome composition primarily to passive dispersal [24]. However, specific bacterial taxa are found in higher abundance in the lungs than expected from oral sources, indicating that both neutral processes and selective pressures shape the lung microbiome [24]. However, these hypotheses are currently based on studies of the lung bacteriome, and whether the dynamic characteristics of the lung virome can be explained through similar mechanisms remains to be explored.

Nevertheless, a handful of studies have explored the virome of healthy LRT. Characterization of the LRT virome in healthy subjects have revealed a high prevalence of members of the *Anelloviridae* family, in addition to a high frequency of bacteriophages [25, 26, 27]. A Korean study by Choi et al. examined the LRT virome by profiling induced sputum samples from 12 healthy individuals using NGS [9]. Their analysis revealed bacteriophages, predominantly *Streptococcus* phage, as the most abundant viral group, followed by herpesviruses comprising 13.4 ± 5.2% of detected viruses. Within the herpesvirus group, *Epstein‐Barr Virus* (EBV) (7.07%), CMV (5.42%), and *Herpes Simplex Virus‐1* (0.93%) were the most prevalent. Intriguingly, a report from the USA also found that *Streptococcus* phage constituted 90% of the DNA virome in one healthy individual, and phage populations vary with the number of bacteria in the host [26]. Remarkably, both studies identified *Streptococcus* phage as a significant component of the healthy LRT virome, regardless of geographic regions. Phages play a significant and direct role in shaping bacterial communities. Bacteria utilize their prophages to eliminate competing bacteria or to regulate the overgrowth of other bacteria sharing the same ecological niche, aiding in their survival and reproduction. However, current research on the pulmonary virome remains limited, with substantial gaps in our understanding of viral diversity, function, and interactions within the lung ecosystem. Further research focused on the healthy lung virome is needed to establish baseline characteristics and better understand its role in maintaining respiratory health.

## 3 Host–Virome–Bacteriome Interactions in the Respiratory Tract

Host–microbe interactions in the respiratory tract occur primarily at mucosal interfaces. Resident microbes help “prime” the immune system locally and systemically, shaping responses in epithelial cells, dendritic cells, and neutrophils. This priming occurs through the sensing of pattern‐recognition receptor (PRR) ligands and microbe‐derived metabolites, some of which can enter the circulation and modulate immunity in distant organs. Compared with the gut, this complex axis remains less well defined in the lung [28, 29]. Nevertheless, accumulating evidence indicates that respiratory multi‐omics provides complementary perspectives on virus–host interactions at the mucosal interface. For example, in adults with early mild to moderate COVID‐19, mucosal transcriptomic data indicated a measurable relationship between viral load and immune alterations, suggesting load‐dependent immune responses [30]. In addition, children with weaker innate interferon (IFN) responses show nasopharyngeal viromes enriched for picornaviruses (mainly rhinoviruses) and bacteriophages, linking virome composition to host antiviral immunity [31]. Moreover, *Siphoviridae* phages correlated negatively with in vitro–induced antibacterial DNA and antiviral cytokines, but positively with inflammatory cytokines induced by lipopolysaccharide, including IL‐6, IL‐8, and tumor necrosis factor (TNF). Together, these patterns suggest that phage abundance may act as a proxy for bacterial host expansion and, context‐dependently, as an ecological readout of mucosal defensive status, rather than merely a marker of inflammatory reactivity [31]. Causal directions, however, remain to be established through longitudinal and interventional studies.

Beyond a multi‐omics framework, a focus on airway viral persistence can further elucidate virus–host interactions. Respiratory viruses have traditionally been viewed as causes of acute disease, with inflammation resolving once infection clears [32]. However, growing evidence indicates that genomic RNA from certain viruses—such as rhinovirus, RSV, and SARS‐CoV‐2—can persist in specific tissues despite clinical recovery [33, 34, 35], supported by immune‐evasion strategies that enable incomplete clearance. Classically, viral spread relies on the release of fully assembled virions into the extracellular space, permitting infection of new target cells [36]. Extracellular virions expose envelope glycoproteins or capsid antigens that act as pathogen‐associated molecular patterns (PAMPs), engaging host sensors and promoting elimination of infected cells [37]. Remarkably, several respiratory viruses, including influenza virus, RSV, HMPV, and parainfluenza viruses, also exploit alternative transmission modes [36]. Through syncytia and intercellular extensions, they mediate direct cell‐to‐cell transfer of virions between adjacent or even distant cells [36]. This route limits exposure to neutralizing antibodies and extracellular PRR surveillance, facilitating tissue‐level RNA persistence and, in some settings, latency‐like states. In parallel, many RNA viruses generate defective viral genomes (DVGs), particularly “copy‐back” forms, which recalibrate innate immunity to balance replication and persistence [32, 38]. Copy‐back DVGs arise when the viral polymerase dissociates from the template and re‐anneals to the nascent strand, producing reverse‐complement duplications of specific sequences; these shorter genomes exhibit a replicative advantage over the full‐length genome and serve as potent inducers of IFNs and survival signaling pathways, thereby supporting persistent infection [38]. In RSV lung infection, DVGs could activate retinoic acid‐inducible gene I‐like receptors (RLRs), driving interferon regulatory factor (IRF) signaling and expression of TNFα, IFNλ, and interferon‐induced protein with tetratricopeptide repeats 1 (IFIT1), which restrain replication while promoting survival of persistently infected cells [39, 40]. Similarly, influenza virus and HMPV can also evoke sustained, low‐level IFN responses through the production of DVGs [38]. Collectively, these mechanisms provide a mechanistic basis for chronic, low‐grade immune activation after apparent clinical resolution, with tissue‐ and host‐context dependence.

It is also important to highlight the complex virus–bacterium interactions within the respiratory microbiome, where both synergistic and antagonistic relationships can shape respiratory tract homeostasis. In vitro studies offer mechanistic clues: *Haemophilus influenzae* upregulates intercellular adhesion molecule‐1 (ICAM‐1) and Toll‐like receptor‐3 (TLR3) on airway epithelial cells, thereby facilitating rhinovirus entry [41]. Meanwhile, *Moraxella catarrhalis* has been reported to downregulate TLR3, potentially compromising antiviral defenses [41]. In virus‐dominated contexts, IAV may further modulate the antibacterial activity of unconventional T cells, paving the way for opportunistic pathogens such as *Staphylococcus aureus* and *Streptococcus pneumoniae* [42]. Extending to multi‐omics readouts, under comparable viral infection backgrounds, higher abundances of *Haemophilus influenzae*, *Streptococcus pneumoniae*, and *Streptococcus salivarius* are associated with stronger host transcriptional signals of immune activation, including upregulation of lymphocyte‐related pathways (B cells, NK cells, and granulocyte‐associated genes) and inflammatory cytokine pathways (notably the IL‐1/IL‐6 axis), indicating functional bacterium–virus interactions [43].

The respiratory phageome constitutes the predominant viral constituency in healthy lungs and likely structures bacterial communities, with downstream effects on immune function and pulmonary health. Airway epithelia, commensal bacteria, and resident bacteriophages operate as a mutually dependent triad that governs respiratory—and potentially systemic—homeostasis [44, 45]. It has been proposed that phages may protect the human host from bacterial infections via a symbiotic phage–host partnership [46]. The respiratory epithelium secretes mucus that is essential for bacterial clearance via mucociliary clearance (MCC) [47]. On mucus‐rich epithelial surfaces, phages are enriched through binding between mucin glycoproteins and Ig‐like domains on phage capsids (the bacteriophage‐adherence‐to‐mucus, or BAM, model) [46], which increases local phage concentration, elevates phage–bacterium encounter rates, and promotes killing of pathogenic bacteria by mucosa‐adherent phages. Consequently, mucus‐rich epithelia both restrict bacterial activity and concentrate phages via mucin–phage Ig‐like interactions, thereby adding an additional antimicrobial defense at mucosal surfaces. Dysfunction of mucus secretion—as in asthma—may impair MCC and disrupt phage–bacterial interactions at the mucosal interface, potentially contributing to dysregulation of mucosal immune homeostasis [45].

**FIGURE 1:**
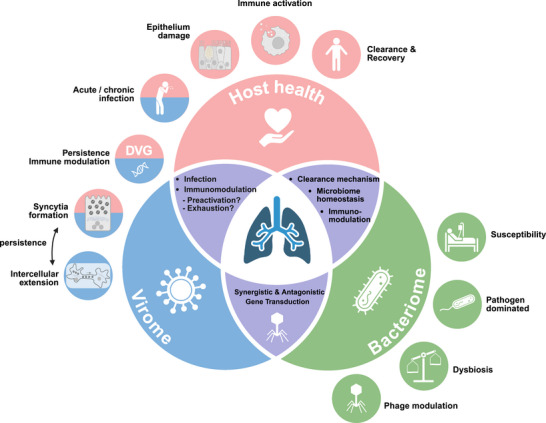
Overview of interactions among the respiratory virome, bacteriome, and host health. The figure depicts how viral and bacterial communities within the respiratory tract collectively influence host health through infection dynamics, immune modulation, regulation of microbial balance, and shaping of disease susceptibility. Key pathways are further discussed in the main text. *Created with BioRender.com*.

Overall, the role of phages in shaping the composition of the respiratory microbiota remains poorly characterized. However, interactions between the gut phageome and bacteriome may offer mechanistic insights [48, 49]. For instance, lytic phages directly kill their bacterial hosts, thereby reshaping community structure and abundance. In addition, after the primary targets are lysed, the resulting reallocation of niches and resources can indirectly influence “non‐susceptible” commensal species—a cascading effect [50]. Through cross‐species propagation and broader network effects, these changes can further remodel microbial interactions and metabolic pathways, ultimately altering the gut metabolome. Phages can also modulate the pathogenic traits of the gut microbiota during lysogeny. For example, the phage transcription factor Cro activates the type III secretion system in *Escherichia coli*, thereby enhancing its virulence [51]. By encoding and horizontally transferring genes for antimicrobial resistance and for carbohydrate and polysaccharide utilization, phages can markedly enhance the proliferation of gut‐resident bacteria [52]. While these mechanisms are well documented in the gut, their applicability to the respiratory mucosa remains to be tested. We hypothesize that analogous phage–bacterium dynamics in the airway could modulate community structure, mucosal metabolite landscapes, and host antiviral set points, warranting longitudinal, multi‐omic, and perturbation‐based studies (Figure 1).

## 4 Respiratory Virome in Acute Respiratory Infections

While the pathogenic effects of most respiratory viruses have been extensively studied, the roles of viruses detected in different parts of the respiratory tract and their mechanisms of persistence remain incompletely understood. Research has shown that the airways of both healthy children and adults host diverse populations of DNA and RNA viruses, along with bacteriophages [9, 26, 53]. Among them, anelloviruses (AVs)—major constituents of the commensal human virome—are universally acquired in infancy and detected throughout the body [54]. Although not definitively linked to specific diseases, AVs levels tend to rise with host immunosuppression, particularly in solid organ transplant recipients [55, 56]. Consistent with this, AVs can comprise about 70% of the eukaryotic virome in plasma and BALF from patients receiving standard antiviral prophylaxis after heart and lung transplantation with postsurgical immunosuppression [57]. Despite their prevalence, AVs are generally not considered pathogenic. In COVID‐19, however, both *Anelloviridae* and *Redondoviridae*—small circular ssDNA virus families—have shown more frequent colonization and higher titers in severe disease, and small circular DNA viruses are found at higher levels in oropharyngeal samples from intubated patients [58]. These findings suggest that typically commensal small circular DNA viruses may serve as markers—or potential modulators—of disease severity under specific clinical conditions.

Patients with acute respiratory tract infections (ARTIs) displayed various airway diversity. A 6‐year follow‐up study of 4407 children with ARTIs found significant respiratory virome changes [59]. Compared to the children who experienced a single ARTI, children with multiple ARTIs had significantly higher Shannon diversity and Chao richness in their respiratory tract, suggesting that repeated respiratory infections may be associated with a more diverse and complex viral community in the airway. Notably, *Propionibacterium* phages were more abundant in multiple ARTIs compared to single ARTI cases. This virome shift correlated with elevated serum tissue inhibitor of metalloproteinases 1 and platelet‐derived growth factor subunits BB levels, and together they strongly predicted multiple ARTIs, highlighting the role of respiratory virome imbalance in recurrent ARTIs. Furthermore, greater viral diversity has been observed in nasopharyngeal samples from children hospitalized with severe acute respiratory infections (SARIs) compared to healthy controls. Samples from SARI cases contained viruses from families such as *Paramyxoviridae*, *Coronaviridae*, *Parvoviridae*, *Adenoviridae*, *Orthomyxoviridae*, *Picornaviridae*, and *Anelloviridae*, whereas healthy children primarily harbored anelloviruses and bacteriophages [16]. However, other studies have drawn different conclusions. A reanalysis of existing oropharyngeal swab DNA sequence data examined the respiratory virome in children with pneumonia [60]. The analysis revealed significantly reduced viral richness and evenness in pneumonia patients compared to healthy controls, demonstrating marked differences in oropharyngeal virome composition between the two groups. Furthermore, in adults with ARTIs, viral diversity and richness in nasopharyngeal swabs showed no difference compared to healthy controls, with *Propionibacterium* phages being the most abundant in both groups [61]. These discrepancies may reflect differences in study populations (e.g., age, immune status, comorbidities), disease characteristics (diagnosis, severity, acute vs. recurrent episodes), sampling sites (nasopharynx vs. oropharynx), and methodological variables (sample processing, extraction, library preparation, sequencing depth/platform, and bioinformatic pipelines). Future studies should adopt standardized pre‑analytical and analytical workflows, report metadata comprehensively, and stratify analyses by population and disease stage to resolve conflicting findings and clarify the role of virome diversity in respiratory disease.

Mechanically ventilated patients also carry diverse viruses in their BALF samples, regardless of infection. Viruses like RSV, *Metapneumovirus*, *Rhinoviruses*, and influenza viruses were found in 30%–50% of samples [62]. A novel DNA virus family, *Redondoviridae*, was identified in critically ill patients’ tracheal aspirates and showed higher loads compared to healthy controls [63]. In addition, redondovirus has been associated with periodontitis in multiple studies, and its levels significantly decrease after effective treatment [63]. Current studies suggest the presence of a common virome signature in the respiratory tract under both healthy and diseased conditions. However, during active infections, the virome burden significantly increases, potentially contributing to severe outcomes. These findings underscore the complexity of the respiratory virome and its dynamic interplay with the host's immune system. A better understanding of the functional roles of these viruses, including their potential pathogenicity or symbiotic effects, is crucial for advancing our knowledge of respiratory diseases.

## 5 Respiratory Virome in Chronic Respiratory Disease

In addition to its role in acute infections, virome research has recently extended into the field of chronic respiratory diseases [64]. This section highlights new insights into respiratory virome characteristics and their potential contributions to the pathogenesis and progression of chronic respiratory diseases.

### 5.1 Chronic Obstructive Pulmonary Disease (COPD)

COPD is a global life‐threatening lung disease characterized by irreversible airflow limitation resulting from inflammation, emphysema, and alveolar damage [65, 66]. Research evidence shows that the pulmonary microbial ecosystem of COPD patients exhibits marked differences from that of healthy subjects regarding both biodiversity and relative abundance, with these distinctions becoming more pronounced during disease exacerbations [67, 68, 69, 70]. Reduced bacterial diversity has been observed in stable COPD patients compared to healthy smokers and non‐smokers, with further declines during acute exacerbations (AECOPD) [70, 71, 72]. Lower bacterial diversity in the lower respiratory tract correlates with worse lung function, more frequent exacerbations, and higher one‐year mortality during AECOPD [69, 73]. These shifts in microbial diversity, indicative of airway dysbiosis, play a key role in COPD pathogenesis via inflammatory and immune pathways.

Viruses also play a significant role in COPD development and progression, yet lung virome studies remain limited compared to bacteriome research. In 2000, a study of 33 COPD patients found rhinovirus infection in 10 of 43 recorded exacerbations [74]. Since then, numerous studies have confirmed the involvement of various viruses, including rhinovirus, influenza, parainfluenza, coronavirus, and RSV [75, 76, 77, 78]. In particular, human rhinoviruses (HRV) were the most commonly identified virus associated with exacerbations, accounting for approximately 40%–60% of detected cases, varying by studies [77, 79, 80]. In addition, Molyneaux et al. [81] showed that HRV infection in COPD patients leads to a sixfold bacterial burden increase and a 16% rise in proteobacteria, particularly pathogenic *Haemophilus influenzae*, identifying rhinovirus as a key trigger for secondary bacterial infections in AECOPD. Additionally, Mallia et al. [82] found that bronchial alveolar lavage cells from COPD patients produced fewer type I and III IFNs when exposed to rhinovirus ex vivo than cells from healthy individuals, suggesting that reduced IFNs production may contribute to virus‐induced AECOPD. However, a multicenter observational study involving 200 COPD patients from Europe and North America, with a follow‐up of up to three years, found that the detection rate of common respiratory viruses in the sputum of stable COPD patients was 15% [76]. In addition, the study found that most respiratory viruses, including coronaviruses, HRV, influenza viruses, human parainfluenza viruses, and RSV, showed a strong association with the occurrence of acute exacerbation events. However, the detection rate of viruses had a weaker correlation with exacerbation frequency, with only coronaviruses and HRV showing a significant link. This suggested that viral infections strongly trigger AECOPD but do not increase susceptibility to repeated exacerbations. Furthermore, a three‐year longitudinal analysis of sputum microbiota in COPD stable patients revealed two groups: the consistent group, characterized by a stable microbiota dominated by *Veillonella, Prevotella, and Streptococcus*; and the variable group, with greater microbiota fluctuations and a higher relative abundance of *Bacillus, Escherichia, Lactobacillus, Moraxella*, and *Staphylococcus* [76]. Notably, Patients with variable sputum microbiota were more prone to frequent viral infections and AECOPD [76]. This suggests a potential relationship between viral infections and microbiota fluctuations, though it remains unclear whether viral infections induce microbiota dysbiosis or pre‐existing microbiota instability increases susceptibility to viral infections, ultimately exacerbating AECOPD risk. Further research is required to elucidate these mechanisms.

### 5.2 Asthma

Asthma is a heterogeneous disease characterized by allergic airway inflammation, remodeling, and hyperresponsiveness [83]. Microbes have long been postulated to play a role in asthma and might also shape its heterogeneity. To date, research on asthma‐associated respiratory microbiota has primarily focused on bacteria, revealing differences in microbiota composition between healthy individuals and asthma patients [84, 85, 86, 87]. In addition to bacterial dysbiosis, growing evidence highlights the importance of the lung virome in asthma pathophysiology. Viruses, particularly respiratory viruses such as HRVs, are key drivers of asthma exacerbations and may interact with the bacterial microbiota to influence disease outcomes [88, 89].

Respiratory viral infections are the triggers most commonly associated with exacerbations [90]. While any viral respiratory infection has the potential to trigger an exacerbation, HRVs are the most frequent cause [91, 92], contributing to approximately 80% of exacerbations in children [93] and 50% in adults [94]. Other viruses, such as RSV [95], human parainfluenza virus [96], and influenza virus [97], are also associated with exacerbations, though their impact is less frequent. The effects of RSV and HRV infections appear especially critical in early life. For example, data from the COAST cohort found that wheezing associated with RSV alone in the first three years of life increased the risk of asthma at age six (odds ratio [OR]: 2.6, 95% CI: 1.0–6.3, P < 0.05), while wheezing associated with HRV alone showed a much stronger association (OR: 9.8, 95% CI: 4.3–22.0, P < 0.05) [93]. These findings suggest that early viral infections may predispose infants and young children to develop asthma later in life.

The mechanisms linking viral infections and asthma include impaired antiviral immunity in affected individuals. Specifically, individuals with asthma exhibit a deficient interferon response to HRV infection, characterized by reduced production of IFN‐α, IFN‐β, and IFN‐λ [98, 99, 100]. This impaired interferon response correlates with higher viral loads, elevated inflammation, airway hyperresponsiveness, and reduced lung function [98]. Such reduced antiviral defense likely exacerbates airway damage, promotes chronic inflammation, and disrupts immune regulation. These deficits may explain why some patients with pre‐existing antiviral immune deficiencies are more susceptible to developing asthma, particularly after recurrent viral infections. Interestingly, viral infections do not act independently in asthma pathogenesis but are often linked to alterations in the upper respiratory microbiome. For example, viral infections commonly trigger microbial imbalances characterized by reduced diversity and dominance of pathogenic bacteria such as *Moraxella*, *Streptococcus*, and *Haemophilus* [86]. Notably, these changes in the microbiome often arise before viral pathogens are detected, suggesting a bidirectional interplay in which bacterial dysbiosis and viral infections amplify each other, increasing asthma severity [86]. These findings underscore the intertwined roles of the viral and bacterial microbiomes in driving asthma pathophysiology.

Beyond single viral infections triggering acute asthma exacerbations, recent research has increasingly focused on how the entire lung virome may influence asthma. A clear shift in virome composition has been observed in asthma. For example, the Predicta study of preschool‐aged children with asthma identified an increased prevalence of eukaryotic viruses, particularly picornaviruses and anelloviruses, compared to healthy controls [17]. This study also revealed that in the combined viral metagenome‐assembled genomes (vMAGs) across all samples, although all assembled bacteriophages were detectable in the asthma group, their abundance was significantly lower compared to the healthy group. Furthermore, in the vMAG subset specific to healthy children, 64.29% were bacteriophages, whereas only 5.88% of asthma‐specific vMAGs were bacteriophages [17]. This indicates that although the virome of asthma patients still harbors bacteriophages, their overall abundance and diversity are markedly reduced. However, the study did not specify the exact types of bacteriophages that were reduced, instead providing an overall description of their significantly decreased abundance and diversity in the asthma group. Researches have linked specific viruses to asthma severity in adults. For instance, Choi et al. [9] found that adult asthma patients, particularly those experiencing exacerbations, exhibited higher levels of herpesviruses such as CMV (24.5%) and EBV (16.9%) compared to healthy controls (5.4% and 7.1%, respectively). CMV and EBV abundance correlated with more severe asthma, reflected in lower Asthma Control Test (ACT) scores, reduced lung function, and increased inflammation. Conversely, bacteriophages, predominantly *Streptococcus* phages, which are abundant in healthy individuals, were significantly reduced in asthma patients, with levels decreasing as asthma severity increased [9]. Notably, the overall abundance of bacteriophages was positively correlated with better asthma control and lung function, reinforcing their potential protective role in maintaining respiratory health [9]. Consistent with the BAM model previously described [45, 46], the immunoregulatory interplay between bacteriophages and airway mucus may support mucosal immune homeostasis, and perturbations of this axis—potentially alongside dysregulated airway microcirculation—could be implicated in aspects of asthma pathogenesis.

### 5.3 Interstitial Lung Disease (ILD)

ILD encompasses a range of disorders characterized by chronic inflammation and lung fibrosis, with idiopathic pulmonary fibrosis (IPF) being the most prevalent and severe [101]. Recent studies have shown that IPF patients exhibit reduced lung microbial diversity and altered microbial composition compared to healthy individuals [102, 103, 104], and dysbiosis in the lung microbiome has been associated with increased inflammation and activation of fibrotic pathways in animal models of pulmonary fibrosis [105].

Beyond the microbiome, the role of the lung virome in IPF remains less well understood, although viruses have long been implicated as potential contributors to fibrosis development. Viral infections can induce pulmonary fibrosis through direct lung damage or by activating immune‐mediated injury [106]. While the wound‐healing response to acute infections is usually self‐limiting, persistent or abnormal healing in the context of chronic infections may result in fibrosis. Furthermore, viral infections stimulate inflammatory cell infiltration and the release of pro‐fibrotic mediators such as transforming growth factor beta (TGF‐β) and interleukins, creating a sustained cycle of immune activation and tissue damage. Several viruses, including human T‐cell leukemia virus [107], herpesviruses [108, 109], influenza virus [110], and coronaviruses [111, 112], have been associated with pulmonary fibrosis. Among these, the human herpesvirus (HHV) family has received the most attention. A meta‐analysis of 20 case‐control studies involving 1287 participants from 10 countries found that persistent or chronic viral infections, including EBV, CMV, HHV7, and HHV8, significantly increase the risk of developing IPF (OR: 3.48, 95% CI: 1.61–7.52, P = 0.001) [109]. However, no significant association was observed between viral infections and acute exacerbations of IPF (OR: 0.99, 95% CI: 0.46–2.12, P = 0.988) [109]. These findings suggest that chronic viral infections may be a potential risk factor for the development of IPF but not for its exacerbation. *Torque teno virus* (TTV) is another virus that has been implicated in IPF, particularly in acute exacerbation (AE‐IPF). BALF analysis identified TTV in 12 out of 43 AE‐IPF cases but detected none in stable IPF patients [113]. Additionally, an independent cohort study demonstrated that IPF patients who tested seropositive for TTV‐DNA had significantly worse survival outcomes [114]. TTV belongs to the *Anelloviridae* family, which is widely present in human blood. Current research suggests that TTV is more likely to indirectly influence disease progression by regulating host immune responses and immune evasion mechanisms rather than acting as a direct pathogenic factor [115]. Therefore, the precise role of TTV in IPF pathogenesis remains unclear, and further studies are required to explore the interactions between TTV, immune modulation, and IPF.

### 5.4 Lung Cancer

**FIGURE 2:**
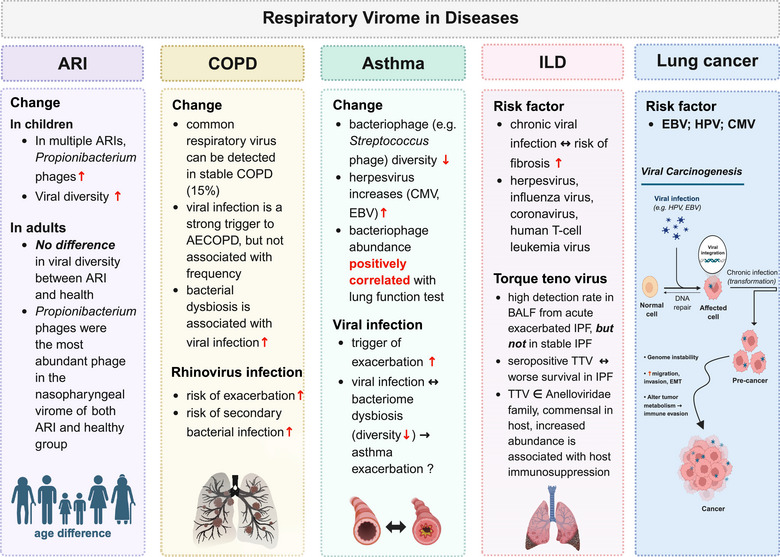
The respiratory virome and diseases. Summary diagram showing the associations between the respiratory virome and various respiratory diseases. *Created with BioRender.com*. Abbreviations: ARI, acute respiratory infection; ILD, interstitial lung disease; IPF, idiopathic pulmonary fibrosis; BALF, bronchoalveolar lavage fluid; EMT, epithelial‐mesenchymal transition.

Viral infections are common causative factors of cancer. For instance, *Hepatitis* B and C viruses are known to cause hepatocellular carcinoma, EBV is associated with nasopharyngeal carcinoma, and *Human Papillomavirus* (HPV) is a well‐established carcinogen in cervical cancer. However, the link between the virome and lung cancer remains under investigation with limited evidence. HPV has been detected in the genital tract as well as the upper respiratory and digestive tracts [116]. A study demonstrated that HPV was detectable in exhaled breath condensate, bronchial brushing, and tumor tissue samples, with infection present in 16.4% of non‐small cell lung cancer (NSCLC) patients but absent in all control subjects [117]. Similarly, a meta‐analysis of 1094 lung cancer patients confirmed a strong association between tumor tissue HPV infection and lung cancer (OR = 5.67, 95% CI: 3.09–10.40, P < 0.001) [118]. Notably, HPV16/18 showed a particularly strong link to lung squamous cell carcinoma, with an odds ratio of 9.78 (95% CI: 6.28–15.22, P < 0.001). These findings suggest a significant pathogenic role for HPV, especially HPV16/18, in lung cancer. EBV is known to cause pulmonary lymphoepithelioma‐like carcinoma [119], but studies have also found EBV DNA and proteins in lung cancer tissues. For example, a study identified EBV in 8% of lung cancer cases and detected BamH1‐A Rightward Frame 1 (BARF1) transcripts in 46% of the EBV‐positive cases [120]. In addition, data from lung cell cultures showed that BARF1 promotes cell migration, invasion, and epithelial‐mesenchymal transition, underscoring the potential role of EBV in lung cancer progression [120]. Furthermore, EBV promotes tumorigenesis through key viral proteins such as LMP1 and LMP2A manipulate glycolysis, glutaminolysis, and lipid metabolism to support viral replication and immune evasion within the tumor microenvironment [121]. These interactions may provide insights for developing therapies targeting viral‐induced metabolic vulnerabilities in EBV‐associated lung cancer. CMV is also detectable in the respiratory tract in patients with lung cancer [122]. Moreover, recent research proposed that CMV‐induced circulating senescent CD8+ T cells (T_8_sen) were associated with worse progression‐free and overall survival in patients treated with immune checkpoint inhibitors, indicating that CMV as the unique viral driver of T_8_sen‐driven resistance to immunotherapy in patients with advanced NSCLC [123] (Figure 2).

## 6 Challenges and Methodological Advances in Respiratory Virome Research

The respiratory virome is difficult to profile due to challenges in obtaining representative samples, intrinsically low biomass, interference from host nucleic acids, and limited high‐quality reference genomes for virome analyses. Many of these issues are tightly linked to sample type and collection method, each with distinct advantages and drawbacks.

Respiratory samples are generally classified as invasive—BALF and tracheal aspirates—or non‐invasive, including sputum (spontaneous or induced) and exhaled breath condensate (EBC). BALF requires bronchoscopy, and tracheal aspirates are typically collected from intubated, critically ill patients; thus, invasive sampling largely targets diseased cohorts and provides little insight into commensals in healthy populations. EBC, obtained by condensing exhaled vapor, is minimally invasive but has not reliably reflected the lower airway microbiome in animal models [124], and no human studies have directly compared EBC with BALF, leaving its suitability for lower airway profiling uncertain. Sputum is easy to obtain but heterogeneous: expectorated material can represent (1) upper airway microbiota, (2) a mixture of upper and lower airways, or (3) predominantly lower airways. The relative contribution from each site is unclear, and sputum collection methods (spontaneous vs. induced) can further influence the results [125]. Consequently, no ideal surrogate exists for studying the lung virome.

Airway samples are low‐biomass and bacteria‐dominated; viruses are less abundant and harder to detect, and many—especially RNA viruses—degrade rapidly without strict preservation. These features complicate sample preparation and library construction. Contamination from external microbial DNA introduced during sampling and processing can exceed the true signal in lower airway specimens, biasing libraries toward exogenous sequences [126, 127]. Host DNA may account for >99% of total nucleic acids, masking viral reads [128]. Additional hurdles include the absence of universal viral marker genes (analogous to bacterial 16S or fungal 18S) and incomplete respiratory viral genome databases, which together impede accurate identification, taxonomic classification, and functional annotation even with advanced sequencing platforms.

Accordingly, enriching virus‐like particles (VLPs) is essential for effective virome sequencing. Although most VLP protocols were developed for gut viromics, they provide a useful framework for respiratory applications [6, 129, 130, 131]. The primary objective of VLP enrichment is to maximize viral particle recovery while minimizing host and bacterial contaminants and excess fluid, thereby increasing VLP concentration and ensuring high‐quality input for downstream nucleic acid extraction. Core approaches include filtration, ultracentrifugation, and chemical enrichment, often combined with enzymatic treatments to deplete free host nucleic acids and improve VLP isolation efficiency.

Filtration employs membranes with defined pore sizes to pass VLPs while excluding larger entities such as bacteria, fungi, and host cells, enabling size‐based separation. It is simple and accessible, commonly using syringe filters of 0.22 µm or 0.45 µm [132, 133], and has been applied to enrich eukaryotic viruses from intestinal samples [6]. However, the selected pore size can significantly influence the efficiency of viral enrichment. For instance, using a 0.22 µm filter may result in the loss of larger viruses [132] and can reduce the yield of viral DNA recovered from human fecal samples by half compared to a 0.45 µm filter [133]. Additionally, filters are prone to clogging by impurities, further reducing throughput and recovery.

Ultracentrifugation (UC) separates VLPs based on particle size, density, and shape under high g‐forces, typically employing density gradients to achieve optimal particle resolution. This established, reproducible method concentrates a broad range of virions and has been used to enrich large quantities of phages from human feces [129]. Nevertheless, the intense centrifugal forces may damage viral particles and compromise biodiversity within the sample. Moreover, this method requires costly, specialized UC equipment and involves complex operations, all of which restrict its broader application.

Chemical enrichment uses reagents that modulate VLP solubility or buoyant density. Commonly used agents include polyethylene glycol (PEG) and cesium chloride (CsCl). PEG precipitates proteins, and because phage capsids are primarily proteinaceous, PEG can efficiently pull phages out of solution. The PEG precipitation technique has long been applied to enrich viral particles across diverse environmental sources [134]. Recently, Shkoporov et al. [130] introduced a targeted PEG‐based protocol for gut phageome enrichment. However, PEG can generate viscous, high–molecular weight residues that are difficult to remove via buffer exchange and may impair VLP recovery [131], further reducing throughput and data yield. CsCl density gradients are another common option [135]. In one study, a workflow combining filtration, DNase treatment, and CsCl density gradient centrifugation was used to isolate VLPs from fecal samples. This approach removed host‐derived DNA more effectively than filtration plus DNase alone but showed marked selectivity against specific phages and lower reproducibility for quantitative analyses [131].

Beyond gut‐standard methods, environmental and wastewater viromics offer complementary strategies. In parallel, (i) pressure‐driven membrane processes—such as ultrafiltration (UF)—use molecular weight cut‐off (MWCO) defined membranes to retain intact virions and long nucleic acids while removing salts and small inhibitors, thereby enabling high separation yields, although insufficient pre‐clarification can lead to membrane fouling and material‐specific adsorption that reduce recoveries [136, 137]; and (ii) Nanotrap particles—micron‐scale, magnetizable hydrogel beads with large planar dye molecules embedded in situ as affinity baits—selectively capture and concentrate targets like host biomarkers, viral nucleic acids, and proteins from complex matrices [137, 138]. A wastewater study comparing UF, Nanotrap enrichment, and PEG reported overall viral recoveries of ∼16%, 64%, and 48%, respectively [137]; sequencing indicated PEG yielded the highest viral diversity and abundance. Yet those protocols processed up to 40 mL per sample—volumes rarely feasible for routine respiratory specimens—so direct transfer to airway workflows requires validation and volume‐scaling optimization. Table 1 summarizes the VLP enrichment methods evaluated here, highlighting their mechanisms, input volume requirements, reported recoveries, and matrix‐dependent limitations.

**TABLE 1:** Comparative evaluation of viral enrichment strategies in metagenomics.

Stratege	Principle	Bias/Selectivity	Cost	Time	Processable reaction volume (single run)	Advantages	Limitations
syringe filter (0.22 or 0.45 µm) [132, 133, 139]	Remove cells/bacteria by pore‐size exclusion while retaining viral particles	Medium: size‐based exclusion biases against giant viruses	$	∼0.5 h	low (1–5 mL)	Fast, inexpensive, and standardized; Good at remove host and bacteria.	1. Prone to clogging; 2. membrane material (PES/PVDF/NC/CA) and pore size determine recovery differences.
Ultrafiltration (30–100 kDa MWCO) [136, 137]	Concentrates viruses via MWCO	Medium: MWCO‐driven selectivity, potential co‐enrichment of exosomes/proteins	$$	0.5–1 h	Medium (0.5–15 mL, device‐dependent)	Volume reduction; Improved sensitivity; Operationally robust and efficient.	Recovery loss due to membrane adsorption
Ultracentrifugation [129]	Size‐/density‐based sedimentation or isopycnic separation	Medium–High: dependent on virion density and morphology	$$$	4–24 h	large (10–50 mL, device‐dependent)	High‐purity VLPs and reduce inhibitors	1. High equipment and time costs; 2. Potential damage to enveloped viruses; 3. Limited throughput.
Nanotrap particles /magnetic beads [137, 138]	Capture and concentrate nucleic acids using functionalized nanoparticles (affinity/electrostatic/hydrophobic interactions)	Medium–High; determined by surface ligands	$$	0.5–1.5 h	ultra‐large (>50 mL)	Fast and easy to operate; Compatible with magnetic separation; Improves qPCR/sequencing positivity rates; Enrichment from low‐titer samples; Suitable for rapid field‐deployable workflows.	1. Dependence on viral lineage and envelope status due to ligand‐driven capture; 2. Potential co‐enrichment of inhibitors; 3. Requirement for elution optimization.
Others: Electrostatic microfiltration [140]	Capture and retention via surface‐charge–mediated electrostatic adsorption during microfiltration	Medium: selective for charged species and surface properties	unknown	∼0.5 h	ultra‐large (>50 mL)	Rapid concentration with anti‐fouling; Reduced non‐specific adsorption; Maintains recovery at relatively high throughput.	1. Not yet commercialized; 2. Compatibility across diverse viral lineages requires validation.

Abbreviations: PES, polyethersulfone; PVDF, polyvinylidene fluoride; NC, nitrocellulose; CA, cellulose acetate; MWCO, molecular weight cut‑off; qPCR, quantitative polymerase chain reaction; VLPs, virus‐like particles; µm, micrometer; mL, milliliter; h, hour.

Current respiratory virome enrichment remains suboptimal. Typical workflows liquefy sputum with dithiothreitol, perform low‐speed centrifugation (6000×g or 8000 rpm for 5 min) and syringe filtration to remove cellular and bacterial debris, followed by followed by DNase, with optional RNase, to eliminate free host nucleic acids and specialized kits to extract viral nucleic acids [9, 139]. Even so, comparative studies show viral sequences comprise only ∼5%–8% of total reads from respiratory samples [139]. By contrast, gut virome samples contain ∼5% VLPs pre‐enrichment, rising to ∼80% after enrichment [131]. This gap largely reflects markedly lower viral loads in airway samples. Developing higher‐yield, low‐bias enrichment tailored to small‐volume respiratory samples is therefore a key priority for future research.

## 7 Emerging Sequencing Technologies in Viromics

In viromics, short‐read sequencing (SRS) remains dominant due to high throughput and low per‐base cost, but its 75–300 bp reads cannot uniquely span low‐complexity repeats, tandem arrays, segmental duplications, and complex structural variants—features that disproportionately drive assembly gaps and missing sequence [141]. Long‐read sequencing (LRS) alleviates these bottlenecks and expands the scope and resolution of studies on human respiratory pathogens, revealing repeat arrays and structural variations that were previously under‐annotated. Two major LRS approaches—single‐molecule real‐time (SMRT) sequencing from Pacific Biosciences (PacBio) [142] and nanopore sequencing from Oxford Nanopore Technologies [143] —now dominate practice. Detailed mechanisms and performance benchmarks are reviewed elsewhere [141, 144, 145]; below is a concise summary of strengths, limitations, and viromics use cases.

SMRT is a single‐molecule, sequencing‐by‐synthesis method. PacBio's platform uses zero‐mode waveguides (ZMWs) to monitor polymerase‐driven strand extension via base‐specific fluorescent pulses [146]. Consequently, read length is tightly coupled to polymerase processivity and activity, which in turn are affected by cumulative laser exposure. Early SMRT applied “continuous long read” runs to produce ultralong reads up to 16 kb, yielding roughly 55–365k reads per SMRT Cell with raw error rates of 13%–15% and run times of ∼0.5–10 h [144]. By contrast, Illumina typically generates 2 × 150 or 2 × 300 bp reads, up to 5–25 billion reads per run at ∼0.1% error rates in <1–3.5 days [144], therefore, early SMRT underperformed Illumina in per‐read accuracy and overall throughput. However, the advent of high‐fidelity (HiFi) sequencing changed this picture: size‐selecting 15–20 kbp inserts enables multiple polymerase passes (typically 7–12), collapsing subreads via circular consensus to yield ∼10–20 kb reads with per‐read accuracies approaching 99.99% (Q40) [147]. These highly accurate long reads improve structural variant discovery and resolution of challenging repetitive regions; nonetheless, deployment remains constrained by instrument and library‐prep requirements and by cost–throughput trade‐offs.

Unlike other third‐generation platforms, nanopore sequencing reads single DNA or RNA molecules in real time without polymerase‐mediated synthesis, sensing nucleotides as they traverse nanopores under an applied voltage [148]. As nucleotides pass through the pore, distinctive ionic current patterns are recorded and computationally decoded into base calls [149]. Standard Oxford Nanopore platforms routinely produce reads an order of magnitude longer than typical SMRT outputs—tens to hundreds of kilobases—with accuracies of ∼87%–98% [145]. Continued improvements in chemistry and base‐calling have raised performance, with recent reports achieving ≥99% accuracy [145, 149]. For virome‐focused work, nanopore sequencing offers real‐time data streams, field‐deployable workflows, direct base‐modification detection, and native RNA sequencing, supporting emergency surveillance and rapid source tracing. Principal drawbacks are higher raw error rates and pronounced homopolymer‐associated biases, motivating hybrid error correction and stringent quality control.

Benchmarking studies have evaluated the impact of these platforms on viral community reconstruction [150]. With single technologies, Illumina assemblies performed best for phage genome recovery. Nanopore‐ and PacBio‐only assemblies underperformed Illumina on genome recovery and error rates, with both metrics varying by assembler. The best Nanopore assembly showed single‐nucleotide polymorphisms (SNPs) and insertions/deletions (INDELs) errors 41% and 157% higher than Illumina‐only assemblies, respectively. Similarly, the best PacBio assemblies exhibited SNP and INDEL error rates 12% and 78% higher than Illumina‐only assemblies, respectively. Overall, the study concluded that combining Illumina and Nanopore reads was most effective, reducing error rates to those of short‐read‐only assemblies; when constrained to a single platform, Illumina remained the preferred option. Looking ahead to viromics applications, hybrid long‐ and short‐read strategies can simultaneously reduce sequencing error rates and recover more comprehensive structural and functional information, thereby substantially improving assembly contiguity and downstream analytical quality.

## 8 Virus‐and Virome‐Oriented Therapeutic Approaches

Progress in virome research—particularly its expansion and increasing standardization—has begun to yield insights that may support the cautious exploration of virus‐ and virome‐oriented interventions across various diseases. For example, under pathological conditions, phage therapy has emerged as one of the most promising strategies for the treatment of refractory bacterial infections, because of its ability to selectively eliminate target bacteria without impacting the host microbiota, compatibility with antibiotics, and low immunogenicity [151]. Evidence from case reports illustrates this potential: during therapy with phage INF, sputum cultures from a patient with cystic fibrosis reportedly shifted from pan‐drug‐resistant *Pseudomonas aeruginosa* to normal respiratory flora, accompanied by notable symptom relief [152]. However, rapid recurrence of *Pseudomonas aeruginosa* upon cessation of phage therapy in the same case underscores intrinsic limitations of phage‐mediated bacterial control and the likelihood that sustained or adjunctive strategies may be required [152]. Currently, multicenter clinical trials are underway globally to assess the safety and efficacy of phage therapy for multidrug‐resistant bacterial infections [153]. In contrast to treatment with whole bacteriophage preparations, phage‑derived endolysins and engineered lysins may provide specific advantages in the control of bacterial infections. Their targets are highly conserved elements of the bacterial cell wall, which likely contribute to the relatively infrequent emergence of resistance. In murine models, local delivery of phage endolysins retained activity in the presence of pulmonary surfactant and conferred protection against *Pseudomonas aeruginosa* lung infection [154].

Recent advances in virome profiling suggest virome‐oriented strategies may extend beyond infectious diseases to immune‐mediated conditions. In saliva from Sjögren's disease (SjD) patients—particularly with higher activity—virome analysis revealed disease‐associated enrichment of eukaryotic viral sequences, with *Vientovirus* more frequently detected [155]. Evidence from follow‐up studies indicates potential molecular mimicry between a *Vientovirus* capsid epitope and SSA/Ro52, accompanied by anti‐SSA/Ro52 responses and partial reproduction of SjD‐like immunologic features in experimental models. Collectively, these observations support a mechanistic model in which viral factors could contribute to the initiation or amplification of autoimmunity via molecular mimicry and, in turn, offer a biological rationale for exploring disease‐targeted precision approaches, such as antivirals or vaccination.

## 9 Conclusions and Future Directions

In this review, we examined the available research on the respiratory virome. Recent studies suggest that the healthy lung is not sterile but may harbor diverse DNA/RNA viruses and bacteriophages. Phages within the mucin‐rich airway mucosa could co‐regulate commensal bacteria and, together with the epithelium, may tune mucociliary defenses and mucosal immune set‐points to help maintain respiratory homeostasis, while some common respiratory viruses might persist at low levels and potentially modulate immunity through longer‐term host interactions. Small circular ssDNA viruses (e.g., *Anelloviridae, Redondovirida*e) in the airways appear to fluctuate with immune tone and could serve as tentative indicators of host immune status. Across acute infection, chronic airway disease, and critical illness, the lung virome may exhibit characteristic shifts in composition and diversity that intersect with mucosal barrier injury, PRR/IFN‐driven innate responses, and the possible breakdown of interdependent microbial networks, collectively potentially amplifying inflammation and tissue damage. These observations are fueling interest in colonization signatures and temporal dynamics as candidate biomarkers for early warning, disease subtyping, severity stratification, and treatment monitoring.

Nonetheless, progress is constrained by predominantly cross‐sectional designs, scarce healthy baselines, and limited mechanistic validation. Causal inference frequently relies on ecosystem‐level perturbations rather than strain‐specific inoculation or depletion, and technical hurdles—low‐biomass sampling, high‐host nucleic acid background, contamination risks, heterogeneous laboratory workflows, and incomplete reference databases—undermine reproducibility and leave substantial virome “dark matter”. To move from correlation to mechanism and translation, priorities are: 1) define stratified healthy baselines and the core lung virome across age, geography, exposure, season, and immune status; 2) implement longitudinal and interventional designs to resolve temporal dynamics and potential causality, integrating host transcriptomics and immune phenotyping; 3) standardize low‐input library preparation, contamination controls, and bioinformatic workflows, alongside high‐quality reference databases and open benchmark datasets; and 4) use integrative multi‐omics to more systematically characterize virome–host–microbe interactions, prioritize potential driver taxa and threshold effects, and lay groundwork for clinically translational virome studies. With these advances, virome‐informed diagnostics, risk models, and targeted interventions could become increasingly feasible, enabling earlier detection, improved patient stratification, and more personalized therapy for respiratory diseases.

## Funding

This work was funded by Natural Science Foundation of China (82341113) and National Key R&D Program of China (2022YFA1304303).

## Conflicts of Interest

The authors declare no conflicts of interest.

## Data Availability

The authors have nothing to report.
